# Newcastle Disease Virus Expressing Cap Gene of Porcine Circovirus Type 2 Confers Protection in Mice and Induced Long-Lasting Neutralizing Antibodies in Pigs

**DOI:** 10.3390/vaccines12111285

**Published:** 2024-11-15

**Authors:** Sohini Dey, Rudhreswaran Murugasamy, Lukumoni Buragohain, Ajai Lawrence D’silva, Jayashree Sarma, Arpita Bharali, Saravanan Ramakrishnan, Mani Saminathan, Nagendra Nath Barman, Vikram N. Vakharia, Madhan Mohan Chellappa

**Affiliations:** 1 Recombinant DNA Laboratory, Division of Veterinary Biotechnology, ICAR-Indian Veterinary Research Institute, Izatnagar, Bareilly 243122, UP, Indiaajailawrencedsilva@gmail.com (A.L.D.); 2 College of Veterinary Science, Assam Agricultural University, Guwahati 781022, AS, Indiajayashreesarma4@gmail.com (J.S.);; 3 Immunology Section, ICAR-Indian Veterinary Research Institute, Izatnagar, Bareilly 243122, UP, India; dearsaromib@yahoo.com; 4 Centre for Animal Disease Research and Diagnosis, ICAR-Indian Veterinary Research Institute, Izatnagar, Bareilly 243122, UP, India; drswamyvet@gmail.com; 5 Institute of Marine & Environmental Technology, University of Maryland, Baltimore County, Baltimore, MD 21202, USA

**Keywords:** Porcine Circovirus 2, Newcastle disease virus, recombinant vector, immune response, mice and pig models

## Abstract

**Background/Objectives**: Porcine Circovirus 2 (PCV2) infection poses significant health and economic challenges to the global swine industry. The disease in pigs leads to lymphoid depletion, resulting in immunosuppression and increased susceptibility to co-infections with other bacterial and viral pathogens. This study evaluated the efficacy of two novel recombinant Newcastle disease virus (NDV) strain R2B vectored vaccines that express the cap gene of PCV2 alone and along with the transmembrane and cytoplasmic tail (TMCT) domains of the NDV F gene. The efficacy of the vaccine candidates was studied in mouse and pig models. **Methods**: Six-week-old BALB/c mice were divided into five groups and immunized intramuscularly three times at 14-day intervals with various vaccine candidates, namely rNDV-R2B-PCVcap-TMCT, rNDV-R2B-PCVcap, and CircoFLEX commercial vaccine, along with controls. Following immunization and PCV2d virus challenge, multiple assays assessed the immune responses in animal trials. In the pig animal trial, pigs were divided into four groups: a control group (PBS), NDV-vectored PCVcap-TMCT group, NDV-vectored-PCVcap group, and CircoFLEX vaccine group. Pigs were immunized intramuscularly twice at 28-day intervals. Blood samples were collected at regular intervals over 70 days to evaluate the humoral and cell-mediated immune responses. **Results**: Both mice and pigs’ trials indicated that the NDV-vectored PCV2 cap-TMCT vaccine candidate elicited superior immune responses. In mice, the rNDV-R2B-PCVcap-TMCT group showed enhanced humoral and cellular immunity, increased PCV2-specific antibody levels, higher CD4+/CD8+ ratio, elevated IFN-γ and TNF-α levels, decreased IL-10 levels, reduced viral loads, and minimal histopathological changes. In pigs, the NDV-vectored PCVcap-TMCT group demonstrated better antibody responses, cytokine profiles (IFN-γ and IL-10), and higher levels of PCV2-specific neutralizing antibodies against the PCV2a, PCV2b and PCV2d genotypes when compared to other groups. **Conclusions**: These findings suggest NDV-vectored PCVcap-TMCT vaccine candidate, expressing the cap gene of PCV2 along with the TMCT domain, offers a promising alternative for protecting against PCV2 infection, potentially addressing the challenges posed by emerging PCV2 strains in the swine industry.

## 1. Introduction

Porcine Circovirus Associated Disease (PCVAD) caused by PCV2 is one of the major disease threats affecting the Indian as well as global swine industry, causing substantial economic losses through decreased efficiency, increased operational expenses, and trade limitations [1]. The disease is characterized by immune suppression due to severe lymphoid depletion of both B and T cells, accompanied by pneumonia, nephritis, and hepatitis with multisystemic involvement [2]. PCV2 is a small, single-stranded DNA virus with approximately 1705 nucleotides in length. It contains four main open reading frames (ORFs), with ORF2 coding for the capsid protein, the primary immunogenic component, and is the basis for most commercial vaccines [3]. To date, researchers have identified eight genotypes of PCV2 (a–h), with PCV2d currently being the most prevalent genotype globally [4]. In India, the overall prevalence of PCV2 is 28.2%, with regional variations across northeastern states represented by PCV2d in 55.64% of cases [5,6,7]. The current prevention strategy for PCV2 relies primarily on vaccination with inactivated or subunit vaccines based on the PCV2a genotype. Commercial vaccines such as Ingelvac CircoFLEX^®^ and Porcilis^®^ PCV have been widely used. However, the emergence of new genotypes, particularly PCV2b and PCV2d, has raised concerns about the cross-protective efficacy of these vaccines. This genotypic shift has resulted in vaccine failures globally, highlighting the need for more effective prevention and control strategies [1].

Recombinant vectored vaccines could be used to overcome the disadvantages of current commercial vaccines, including improved efficacy, broader protection, multivalent potential, and the ability to induce long-lasting immunity. Additionally, they would be effective in young pigs even in the presence of maternal antibodies and can be produced cost-effectively. Newcastle Disease Virus (NDV) has emerged as a promising viral vector [8] for vaccine development against various porcine viral diseases caused by the Nipah virus [9], African swine fever virus [10], Japanese Encephalitis virus [11], and Porcine Reproductive and Respiratory Syndrome virus [12]. Its appeal stems from its ease of genetic manipulation, high virus yield, stable foreign gene expression, genomic stability, and ability to induce strong immune responses in non-avian species like pigs [13,14]. It has also been noted that the transmembrane and cytoplasmic tail (TMCT) domain of the fusion protein of NDV plays crucial roles in viral pathogenesis and immune response by anchoring fused proteins, ensuring proper antigen display, facilitating protein stability, and enhancing immune recognition [15,16]. Studies on the pathogenesis of PCV2 infection to evaluate vaccine candidates have been carried out in mouse models due to its ease of management before testing in pig models [17,18]. In this context, the present study describes the construction, characterization, and immunological evaluation of two live viral vaccine constructs using NDV as a vector containing the cap gene of PCV2 in mice and pig models.

## 2. Materials and Methods

### 2.1. Virus and Cell Line

An Indian isolate of the virulent PCV2d genotype virus was obtained from the College of Veterinary Science, Assam Agricultural University, Khanapara (CVSc, AAU, Guwahati, India) grown in PK-15 cell line, and its nucleotide sequence is available under the accession number MN266483. Characterized Newcastle disease virus strain R2B was rescued in our laboratory [8]. Vero and PK-15 cells were obtained from the National Centre for Cell Sciences (NCCS), Pune, India, and were maintained in Medium 199 supplemented with 10% heat-inactivated fetal bovine serum maintained at 37 °C with 5% CO_2_.

### 2.2. Generation of Recombinant NDV Containing the Cap Gene of PCV2

**Figure 1:**
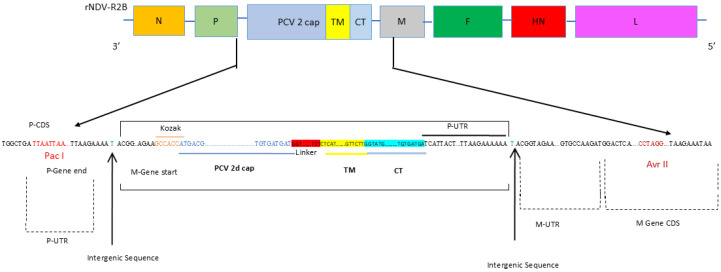
Schematic diagram showing the construction of pNDV-R2B infectious clone harboring the cap gene PCV2. The transcription cassette of PCV2 cap gene was connected with a GS linker to the transmembrane domain and cytoplasmic tail (TMCT) of the F protein of NDV and cloned between the P and M genes that are flanked by Pac I and Avr II restriction sites. P CDS: P gene coding sequence; P-UTR: P gene non-coding region; M-UTR: M gene non-coding region; M Gene CDS: M gene coding sequence.

The pNDV-R2B plasmid and the three support plasmids pNP, pP, and pL were available in the laboratory. Two constructs of the transcription cassette containing the cap gene of PCV2 were designed with mutations including L59A, G191E, and K deletion at 235 positions. The first construct with these three mutations was named as PCVcap. In the second construct, the mutated PCVcap gene was connected with a GS linker to the transmembrane domain and cytoplasmic tail (TMCT) of the F protein of NDV, and it was named PCVcap-TMCT. The PCVcap-TMCT sequence was codon optimized for mammalian host expression. At the 3′ ends of both the constructs, extra nucleotides were added to adhere to the paramyxovirus genome’s “rule of six”. Both the transcriptional cassettes, PCVcap and PCVcap-TMCT, were individually inserted into the P and M genes of full-length infectious clone by directional cloning, using the restriction sites PacI and AvrII (Figure 1). The recombinant plasmids, namely pNDV-R2B-PCVcap and pNDV-R2B-PCVcap-TMCT, were transformed into NEB^®^ Stable Competent Escherichia coli (New England Biolabs Inc., Ipswich, MA, USA). The recombinant viruses, namely rNDV-R2B-PCVcap and rNDV-R2B-PCVcap-TMCT, were rescued in Vero cells, as described previously [8,17].

### 2.3. Characterization of the Virus

The growth kinetics of rescued viruses rNDV-R2B-PCVcap, rNDV-R2B-PCVcap-TMCT, and rNDV-R2B were studied using a multistep growth curve analysis in Vero cells. Viruses were inoculated at an MOI of 0.01 into Vero cells cultured in six-well plates at 37 °C. Supernatants were collected every 12 h over 72 h, and viral titres were measured using the median tissue culture infective dose (TCID50) method. Through fluorescence assay, the recombinant virus (0.01 MOI) infected Vero cells were visualized after 48 h of infection using a Fluroview FV100 confocal microscope (Olympus, Tokyo, Japan), as described [19]. Both the recombinant viruses containing the cap gene of PCV2 were purified using ultra-centrifugation method, as described previously [20]. Through Western blotting, the purified virus was characterized for the presence of NDV and cap protein of PCV2 using anti-NDV polyclonal sera (Abcam, Cambridge, MA, USA) and anti-PCV2 polyclonal antibody (GeneTex, Irvine, CA, USA).

The biological characterization of the recombinant virus was evaluated by mean death time (MDT) and intra-cerebral pathogenicity index (ICPI) analysis as per standard procedures [21].

### 2.4. Confirmation of Virus Stability

The stability of the recombinant viruses was confirmed by serially passaging the viruses in Vero cells. After every 10th passage, viral genomic RNA was extracted from the infected tissue culture fluid and used for cDNA synthesis followed by RT-PCR to verify the amplification using primers for NDV and PCV2 viruses. The amplicons were sequenced to confirm the presence of respective genes.

### 2.5. In-Vivo Animal Experimental Studies

Immunological evaluation of the rNDV-R2BPCVcap-TMCT and rNDV-R2B-PCVcap was carried out in BALB/c mice and pigs after the institute’s animal ethics committee approval. A total of seventy-five female BALB/c mice (5 weeks old), obtained from the National Institute of Nutrition (NIN), Hyderabad, India, were divided randomly into five groups (n = 15). At 6 weeks of age, the mice in the control group were injected with PBS intramuscularly (IM), and the mice in second, third, and fourth groups were inoculated with 3 × 10^8^ TCID50 each of rNDV-R2B-PCVcap-TMCT, rNDV-R2B-PCVcap, and rNDV-R2B, respectively, and the fifth group with 100 μL of Ingelvac CircoFLEX vaccine through IM route. Two booster doses were administered in mice at 14-day intervals following primary vaccination. Further, the mice were challenged 14 days after the second booster with 2 × 10^5^ TCID50 of the PCV2d virus via the intraperitoneal route. Sera samples (n = 5/group) were collected from all mice at 0, 14, 28, and 42 days post-immunization (dpi) and 7 and 14 days post-challenge (dpc). At 42 dpi, 7 and 14 days dpc, five mice from each group were sacrificed to assess cell-mediated immunity. For pig immunization, 25 8-week-old crossbred pigs were procured and housed at the CVSc, AAU under controlled conditions with proper ventilation, access to premixed feed, and clean water. The pigs were randomly divided into five groups (n = 5) and three groups were inoculated each with 9 × 10^8^ TCID50, rNDV-R2B-PCVcap-TMCT, rNDV-R2B-PCVcap, and rNDV-R2B, respectively. The fourth group was inoculated with Ingelvac CircoFLEX vaccine and the fifth control group was immunized with 2 mL of PBS by IM route. The pigs were boosted on the 28th day post-primary immunization with the same preparations. Sera samples (n = 5/group) were collected from the ear vein before vaccination and at 7, 14, 21, 28, 35, 42, 56, and 70 days post-vaccination. The pigs were observed closely for one week after each dose of immunization for any clinical signs or adverse reactions. However, no adverse reaction was observed except for a mild rise in body temperature in a few pigs, which subsided after 72–96 h.

### 2.6. Evaluation of Humoral Immunity in Mice and Pigs

#### 2.6.1. Enzyme-Linked Immunosorbent Assay

Mice and pig sera samples were tested for PCV2-specific antibodies by enzyme-linked immunosorbent assay (ELISA). For mice sera, the 96-well plate was coated with recombinant cap protein [22] for detecting the PCV2-specific antibodies in the vaccinated mice. A cut-off value of 0.22 was fixed by prior optimization, and the values below were considered negative. The anti-PCV2 antibodies in the vaccinated pig sera were estimated using a commercial indirect-ELISA kit (Porcine Circovirus Type 2 Antibodies ELISA Kit, Elabscience, Houston, TX, USA) as per the instructions; the sera samples were diluted at a 1:39 ratio. The antibody titre was expressed in S/P ratio and the ratio ≥0.2 was regarded as positive; otherwise, it was considered negative.

#### 2.6.2. Serum Neutralization Test

The mice sera were heat-inactivated and serially diluted, then incubated with 200 TCID_50_ of PCV2 virus for 60 min at 37 °C. The serum–virus mixture was added to PK-15 cell monolayers in a 96-well plate. After 48 h, cells were fixed, permeabilized, and incubated with an anti-PCV pig antibody (1:500). After washing, HRP-conjugated anti-pig antibody (1:3000) was added. Following washing, DAB substrate (Sigma Aldrich, St. Louis, MI, USA) was added, incubated for 10 min, and the cells were examined. Then, the neutralizing titre was calculated as described earlier [23].

The virus neutralization and cross-neutralization antibody titres were determined in the pig sera against genotypes PCV2d, PCV2a, and PCV2b (available in the repository of ADMaC Laboratory, College of Veterinary Science, Khanapara, Guwahati, Assam, India) by indirect immunofluorescence assay, as described earlier [24,25]. The serum samples (50 μL) of days 14, 35, and 70 (n = 3) were serially diluted two-fold from 1:2 to 1:4096 and incubated with 50 μL of 200 TCID_50_ of PCV2d, PCV2a, and PCV2b viruses at 37 °C for 1 h with 5% CO_2_. The serum–virus mixtures were inoculated in duplicate to a monolayer of PK-15 cells cultured in 96-well plates having 80% confluence and incubated for 72 h at 37 °C. Finally, the virus-neutralizing antibody titres were determined, as described earlier.

### 2.7. Evaluation of Cell-Mediated Immunity

For mice, cell-mediated immune response was evaluated by antigen-specific lymphocyte transformation test at 42 dpi, and 7 and 14 dpc. Similarly, the immune cells of the vaccinated and the control groups were also analyzed by flow cytometry (BD Biosciences, San Jose, CA, USA) to determine the levels of CD4+ and CD8+ T-lymphocyte cells. IFN-γ and IL-10 levels were measured in pg/mL using mouse-specific ELISA kits (Cloud-clone Corporation, Katy, TX, USA) at 42 dpi, and 7 and 14 dpc in all the groups. CMI responses in vaccinated pigs were measured using porcine-specific ELISA kits (Puregene, Jaipur, India), as per the manufacturer’s instructions, at 0, 7, 28, 35, and 56 dpi. The concentration of both IFN-γ and IL-10 were estimated from the standard curve ranging from 60 to 960 pg/mL and 20 to 320 ng/L for IFN-γ and IL-10 cytokines, respectively.

### 2.8. Challenge Studies

Viral load quantification and immunohistochemistry along with histopathological studies were conducted at 7 and 14 dpc in the sacrificed mice (n = 5). The viral load in lungs and inguinal lymph nodes were quantified by real-time PCR using the ORF1 gene of PCV2 virus [26]. A standard curve was used for quantifying the viral genomic copy number. For histopathological analysis, the microscopic lesions of lung, lymph nodes, spleen, liver, and heart tissues of mice at 14 dpc were used. Similarly, at 14 dpc, immunohistochemistry of the inguinal lymph nodes of the challenged mice were examined using PCV2 polyclonal antibody (GeneTex, Irvine, CA, USA).

### 2.9. Statistical Analysis

Statistically significant differences in data from all the groups at different time points were evaluated by two-way ANOVA and the *p* < 0.05 value was considered significant for all the analyses. Statistical analysis was conducted using Prism 8.0.2 (Graph-pad Software Inc., San Diego, CA, USA).

## 3. Results

### 3.1. Stability of Recombinant Virus

Sequence analysis of RT-PCR products obtained from viral RNA isolated from the infected cell culture supernatants confirmed the intactness of NDV junctions and the PCV cap gene in the transcription cassettes, along with the TMCT domain in the rNDV-R2B-PCVcap-TMCT virus even after 10, 20, 30, and 45 passages.

### 3.2. Molecular and Biological Characterization of Recombination Viruses

**Figure 2:**
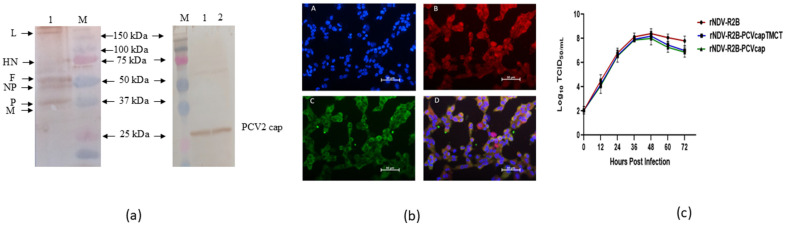
Characterization of the rescued recombinant viruses in vitro. (**a**) Western blot analysis of cell lysate of Vero cell infected with rNDV-R2BPCVcap-TMCT viruses. (i) Position of NDV specific proteins, namely L, HN, F, NP, P and M (left) and (ii) capsid protein of PCV2 and PCV2-TMCT of approximately 27-kDa (right), along with the marker proteins as indicated. Lane M: Precision plus protein standard dual color marker (Bio-Rad, Hercules, CA, USA). (**b**) Demonstration of replication of rescued recombinant rNDV-R2B-PCVcap-TMCT virus in Vero cells by fluorescent microscopy (×40) (**A**) Cells stained with DAPI; (**B**) Cells stained with anti-NDV antibody and secondary antibody (anti-chicken IgG labelled with Alexa Fluor 568); (**C**) Cells stained with anti-PCV antibody and secondary antibody (anti-rabbit FITC) (**D**) Merger of all three frames. (**c**) Multistep growth kinetics of recombinant viruses in Vero cells. Monolayer of Vero cells were infected at 0.01 MOI for rNDV-R2B, rNDV-R2B-PCVcap-TMCT and rNDV-R2B-PCVcap independently. At every 12-h interval until 72 h, the viral titres were determined by limiting dilution assay and calculated as TCID_50_ by Reed and Muench method.

Western blot analysis of the Vero cell culture supernatants containing the rNDV-R2B-PCVcap and rNDV-R2B-PCVcap-TMCT viruses, collected 72 h post-infection (hpi), revealed the presence of NDV proteins and the 27-kDa cap protein of PCV2 (Figure 2a). Moreover, at 48 hpi, the Vero cells infected with recombinant viruses showed the presence of bright green and red fluorescence, confirming the presence of PCV cap and NDV proteins, respectively (Figure 2b). The MDT score of the recombinant viruses was 78 h and the ICPI scores were 1.2. Among the three viruses, rNDV-R2B maintained a slightly higher titre by multi-cycle growth kinetics study, particularly during the peak and late phases, compared to rNDV-R2B-PCVcap-TMCT and rNDV-R2B-PCVcap, which exhibited nearly identical growth patterns at 48 h (Figure 2c). The 45th passage titres of rNDV-R2B-PCVcap-TMCT and rNDV-R2B-PCVcap were 3.8 × 10^9^ TCID50/mL and 3.4 × 10^9^ TCID50/mL, respectively.

### 3.3. Immunogenicity Studies of the Recombinant Viruses as Vaccine Candidates in Mice and Pigs

#### 3.3.1. Immunization with Recombinant Viruses Induce Protective Immune Response in Mice

**Figure 3:**
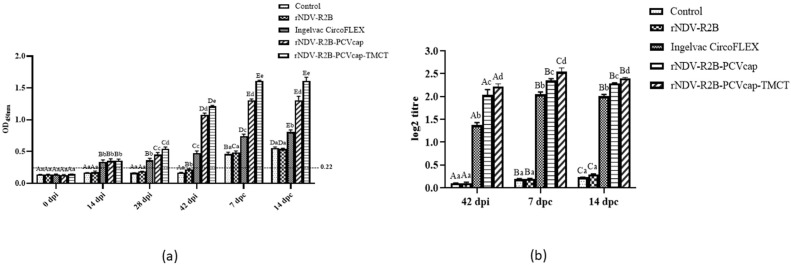
Estimation of PCV2-specific antibody responses in vaccinated and control mice (n = 5/group) by ELISA and SNT. (**a**) Sera samples were collected at regular intervals and tested for anti-PCV2 antibody. The O.D. values > 0.22 were considered positive for anti-PCV2 antibodies. Bars (Mean ± SE) indicate the representative data of a single experiment. Data with different small letters superscript indicate treatment effect (*p* < 0.05) and different capital letters superscript indicate time effect (*p* < 0.05). (**b**) Level of neutralizing antibodies against PCV2 in sera of immunized mice (n = 5/group) are shown as log2 titre values at 42 dpi and 7 and 14 dpc. Bars (Mean ± SE) indicate the representative data of a single experiment. Data with different small letters superscript indicate treatment effect (*p* < 0.05) and different capital letters superscript indicate time effect (*p* < 0.05).

Estimation of PCV2-specific antibodies was studied at 0, 14, 28, and 42 dpi, and at 7 and 14 dpc by ELISA (Figure 3a). The rNDV-R2B-PCVcap-TMCT group showed significantly higher antibody titres from 28 dpi onwards compared to rNDV-R2B-PCVcap and the Ingelvac CircoFLEX groups, while unvaccinated control and rNDV-R2B groups remained low. At 7 and 14 dpc, the rNDV-R2B-PCVcap-TMCT group showed the highest antibody response, followed by rNDV-R2B-PCVcap and Ingelvac CircoFLEX, while the unvaccinated control and rNDV-R2B groups showed minimalistic responses.

For SNT, the neutralizing antibodies were expressed as the reciprocal of the highest dilution of serum that gave positivity in immunoperoxidase assay, as shown in Figure 3b. At 42 dpi, the rNDV-R2B-PCVcap-TMCT group had significantly higher neutralizing antibody titres compared to the other vaccinated groups (*p* < 0.05). By 7 and 14 dpc, the titre increased, with the rNDV-R2B-PCVcap-TMCT group maintaining the strongest response, followed by the rNDV-R2B-PCVcap and Ingelvac CircoFLEX groups.

**Figure 4:**
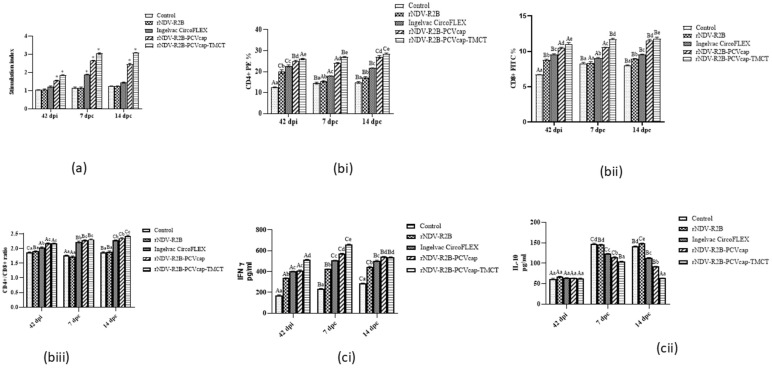
Estimation of cell mediated immune responses in immunized mice at 42 dpi, 7 and 14 dpc. (**a**) Mice splenocytes from vaccinated and control groups (n = 5/group) were stimulated with PCV2 cap protein. Antigen specific lymphocyte proliferation response was measured and expressed as stimulation index. Bars (Mean ± SE) indicate the representative data of a single experiment and * indicates statistical significance (*p* < 0.05). (**b**) Flow cytometric analysis for (**i**) CD4+ cells %, (**ii**) CD8+ cells % and (**iii**) CD4+/CD8+ cells ratio in different vaccinated groups (n = 5/group). Bars (Mean ± SE) indicate the representative data of a single experiment. Data with different small letters superscript indicate treatment effect (*p* < 0.05) and different capital letters superscript indicate time effect (*p* < 0.05). (**c**) Induction of cytokine response namely; (**i**) IFN-γ and (**ii**) IL-10 in control and vaccinated mice (n = 5/group) at 42 dpi, 7 and 14 dpc by ELISA. Values are represented in picogram/mL. Bars (Mean ± SE) indicate the representative data of a single experiment. Data with different small letters superscript indicate treatment effect (*p* < 0.05) and different capital letters superscript indicate time effect (*p* < 0.05).

In the lymphocyte proliferation test, the PCV2 cap protein-specific lymphocyte proliferation was significantly higher at 42 dpi, and 7 and 14 dpc, in the vaccinated compared to control and rNDV-R2B groups (Figure 4a). The rNDV-R2B-PCVcap-TMCT and rNDV-R2B-PCVcap groups demonstrated the highest antigen-specific cell proliferation, followed by the Ingelvac CircoFLEX group, while the control and rNDV-R2B groups showed significantly lower values (*p* < 0.05).

The percentages of CD4+ and CD8+ T-lymphocytes and the CD4+/CD8+ T-cell ratios were evaluated at 42 dpi, and 7 and 14 dpc using splenocytes by Flow cytometry assay. The rNDV-R2B-PCVcap-TMCT group showed significantly higher CD4+ (Figure 4(bi)) and CD8+ (Figure 4(bii)) T-lymphocytes percentages and a balanced CD4+/CD8+ (Figure 4(biii)) T-cell ratio compared to other groups, followed by the rNDV-R2B-PCVcap and Ingelvac CircoFLEX groups (*p* < 0.05).

The cytokine analysis for IFN-γ levels revealed that at 42 dpi and 7 and 14 dpc, the rNDV-R2B-PCVcap-TMCT group exhibited significantly higher IFN-γ levels (*p* < 0.05) when compared to other groups (Figure 4(ci)). Both rNDV-R2B-PCVcap and Ingelvac CircoFLEX groups showed significantly elevated levels compared to the rNDV-R2B and control groups. On the other hand, at 42 dpi, the IL-10 expression exhibited low levels. However, at 7 and 14 dpc, IL-10 levels significantly increased (*p* < 0.05) in the control and rNDV-R2B groups compared to the rNDV-R2B-PCVcap-TMCT, rNDV-R2B-PCVcap, and Ingelvac CircoFLEX groups (Figure 4(cii)).

#### 3.3.2. Estimation of PCV2 Viral Load in Challenged Mice

**Figure 5:**
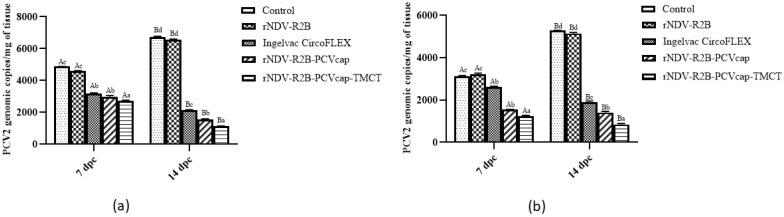
Viral load of PCV2 in (**a**) lungs and (**b**) inguinal lymph node of control and vaccinated mice (n = 5/group) post-challenge at 7 and 14 dpc. Bars represent mean values, and error bars indicate standard deviation. Data with different small letters superscript indicate treatment effect (*p* < 0.05) and different capital letters superscript indicate time effect (*p* < 0.05).

Quantification of viral load, at 7 and 14 dpc in lungs (Figure 5a) and inguinal lymph node (Figure 5b) using real-time PCR demonstrated that the rNDV-R2B-PCVcap-TMCT group had the lowest PCV2 genomic copies (*p* < 0.05) when compared to other groups, followed by rNDV-R2B-PCVcap and Ingelvac CircoFLEX groups (*p* < 0.05). The control and rNDV-R2B groups had the highest viral loads, with levels significantly increased at 14 dpc.

**Figure 6:**
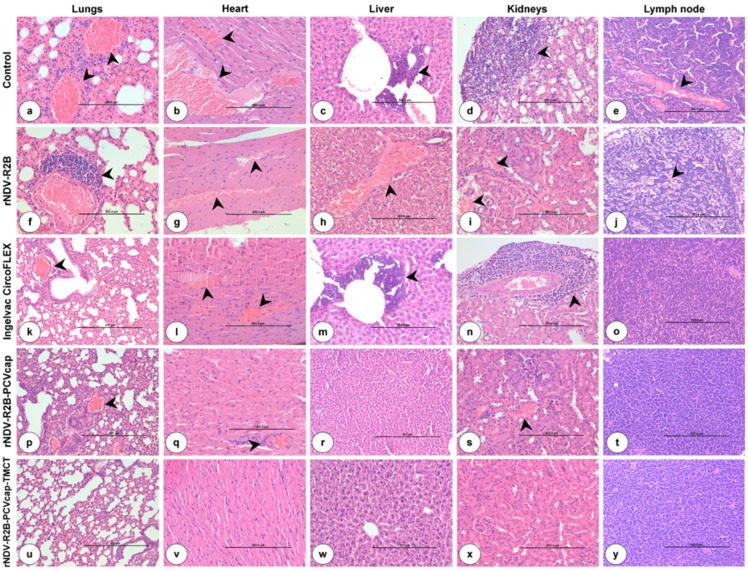
Histopathological lesions in internal organs of mice at 14 days post challenge (dpc). Control (**a**–**e**), rNDV-R2B (**f**–**j**), Ingelvac CircoFLEX (**k**–**o**), rNDV-R2B-PCV (**p**–**t**), and rNDV-R2B-PCV-TMCT (**u**–**y**). (**a**) Lungs showed interstitial pneumonia with thickening of interalveolar spaces and marked congestion (arrow). H&E ×20. (**b**) Heart showed marked dilatation and congestion of myocardial blood vessels (arrow). H&E ×20. (**c**) Liver showed marked infiltration of inflammatory cells in portal triad (arrow). H&E ×20. (**d**) Kidneys showed marked infiltration of mononuclear cells (arrow). H&E ×20. (**e**) Lymph node showed congestion of blood vessels in cortex (arrow). H&E ×20. (**f**) Lungs showed moderate perivascular infiltration of lymphoid cells (arrow). H&E ×20. (**g**) Heart showed moderate to marked congestion of blood vessels (arrow). H&E ×20. (**h**) Liver showed marked congestion of blood vessels (arrow). H&E ×20. (**i**) Kidneys showed congestion and hemorrhages (arrow). H&E ×20. (**j**) Lymph node showed marked lymphoid depletion in cortex (arrow). H&E ×20. (**k**) Lungs showed mild to moderate thickening of interalveolar spaces and congestion (arrow). H&E ×20. (**l**) Heart showed mild to moderate congestion of myocardial vessels (arrow). H&E ×20. (**m**) Liver showed moderate infiltration of inflammatory cells in portal triad (arrow). H&E ×20. (**n**) Kidneys showed moderate infiltration of mononuclear cells (arrow). H&E ×20. (**o**) Lymph node showed mild lymphoid hyperplasia. H&E ×20. (**p**) Lungs showed mild congestion (arrow) and thickening of interalveolar spaces. H&E ×20. (**q**) Heart showed mild congestion of blood vessels (arrow). H&E ×20. (**r**) Liver showed mild congestion. H&E ×20. (**s**) Kidneys showed mild congestion (arrow). H&E ×20. (**t**) Lymph node showed moderate lymphoid hyperplasia. H&E ×20. (**u**) Lungs showed normal alveoli lined by flattened squamous type I pneumocytes. H&E ×20. (**v**) Myocardium showed normal branching cardiac fibers. H&E ×20. (**w**) Liver showed normal central vein with radiating cords of hepatocytes. H&E ×20. (**x**) Kidneys showed normal glomeruli and renal tubules in cortex. H&E ×20. (**y**) Lymph node showed marked lymphoid hyperplasia in cortex. H&E ×20.

**Figure 7:**
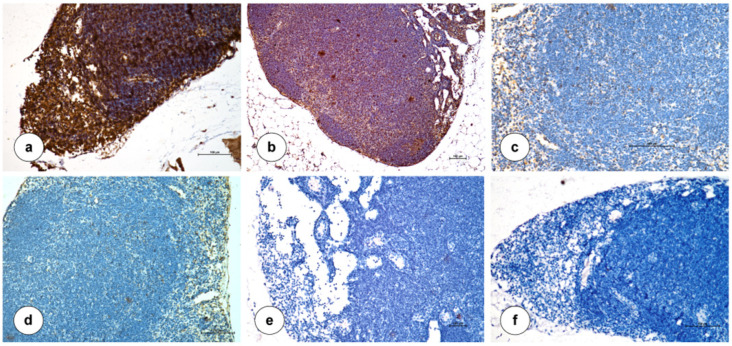
Demonstration of PCV2 antigen in inguinal lymph node of mice at 14 days post challenge (dpc). (**a**) Unvaccinated and infected control group showed marked (+++) immunolocalization of PCV2 antigen. (**b**) rNDV-R2B group showed moderate (++) to marked (+++) immunolocalization of PCV2 antigen. (**c**) Ingelvac CiroFLEX group showed moderate (++) immunolocalization of PCV2 antigen. (**d**) rNDV-R2B-PCVcap group showed mild (+) immunolocalization of PCV2 antigen. (**e**) rNDV-R2B-PCVcap-TMCT group showed absence of immunolocalization of PCV2 antigen. (**f**) Negative control showed no positive immunoreaction for PCV2 antigen. IP-DAB-MH ×20.

Further, the histopathological examination of various organs of mice at 14 dpc showed (Figure 6) severe pathological lesions in the unvaccinated and infected control group and moderate to severe lesions in the rNDV-R2B group. In these groups, lungs showed interstitial pneumonia with thickening of interalveolar spaces due to accumulation of edematous fluid, hemorrhages, and infiltration of inflammatory cells. The heart showed marked dilatation and congestion of myocardial blood vessels. The liver showed marked congestion of the central vein, infiltration of inflammatory cells, especially neutrophils in the portal triad, and necrosis of hepatocytes. Kidneys showed marked congestion of blood vessels, degeneration of tubular epithelial cells, and marked perivascular infiltration of mononuclear cells, especially lymphocytes. Lymph nodes and spleen showed lymphoid depletion with apoptosis of lymphocytes. The uterus showed an accumulation of inflammatory cells, especially neutrophils and macrophages, in their lumen and lamina propria with degenerative changes of mucosal epithelial cells. Ovaries showed degeneration of ovarian follicles with marked congestion of blood vessels. The Ingelvac CircoFLEX group showed mild to moderate histopathological lesions. The rNDV-R2B-PCVcap group showed mild histopathological lesions in all the examined organs, and lymphoid organs showed moderate lymphoid hyperplasia. The rNDV-R2B-PCVcap-TMCT group showed normal histological architecture in the organs and lymphoid organs showed marked lymphoid hyperplasia, indicating sustained protection. Immunohistochemistry of the inguinal lymph node showed (Figure 7) high PCV2 antigen-positive cells in the unvaccinated and infected control and rNDV-R2B groups. A moderate number of PCV2 positive cells were noticed in the Ingelvac CircoFLEX group and a low number of PCV2 positive cells were noticed in the rNDV-R2B-PCVcap group. The rNDV-R2B-PCVcap-TMCT group showed very few to no positive cells. Negative antibody control showed no positive immunoreaction for PCV2 antigen.

#### 3.3.3. Immunization with Recombinant Virus Induces Long-Lasting Immune Response in Pigs

**Figure 8:**
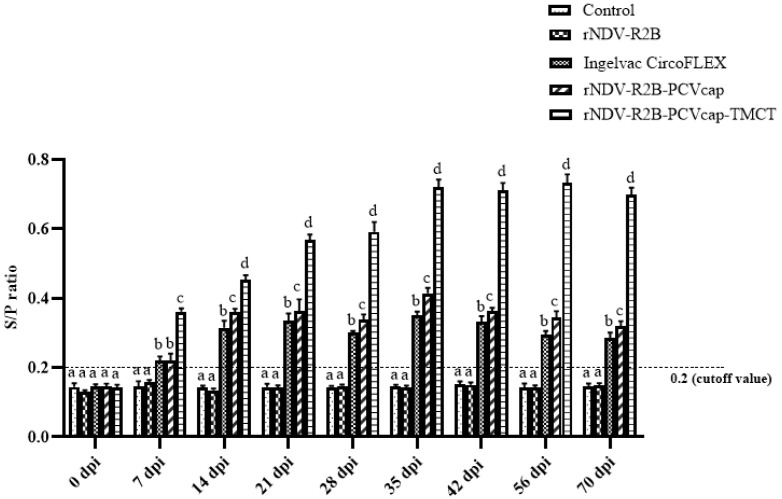
PCV2-specific antibody responses measured by indirect ELISA in pigs of different groups (n = 5/group) from 0 dpi through 70 dpi. The cutoff value for positive antibody response is indicated at 0.2. Bars (Mean ± SE) indicate the representative data of a single experiment. Data with different small letters superscript indicate treatment effect (*p* < 0.05).

Sera samples from all five groups of pigs were screened for PCV2-specific antibodies over 70 dpi. At 0 dpi, all groups tested negative for antibodies by ELISA. By 7 dpi, seroconversion was detected in the rNDV-R2B-PCVcap-TMCT, rNDV-R2B-PCVcap, and Ingelvac CircoFLEX groups (*p* < 0.05), while the control and rNDV-R2B groups remained negative. By 14 dpi and until 70 dpi, the rNDV-R2B-PCVcap-TMCT group had the highest antibody titres, followed by rNDV-R2B-PCVcap and Ingelvac CircoFLEX. The control and rNDV-R2B groups showed values below the cutoff S/P ratio (Figure 8).

**Figure 9:**
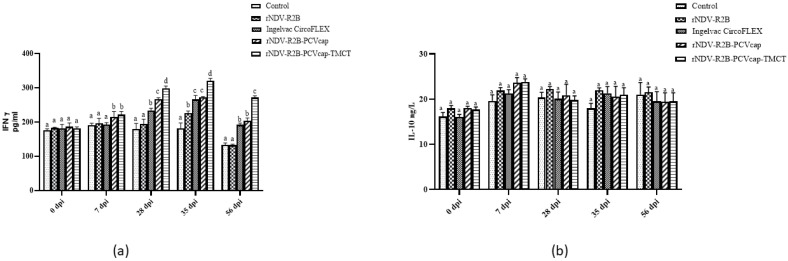
The cytokines (**a**) IFN-γ in pg/mL and (**b**) IL-10 in ng/L levels were analyzed by enzyme linked immunosorbent assay from all the groups (n = 5/group) at 0, 7, 28, 35 and 56 dpi. Bars (Mean ± SE) indicate the representative data of a single experiment. Data with different small letters superscript indicate treatment effect (*p* < 0.05).

The cytokine estimation for IFN-γ levels (pg/mL) by ELISA in pigs showed that at 0 and 7 dpi, all groups had basal levels. By 28 dpi, the rNDV-R2B-PCVcap-TMCT group had the highest IFN-γ levels (*p* < 0.05), followed by rNDV-R2B-PCVcap and Ingelvac CircoFLEX. All three groups had significantly higher levels than the rNDV-R2B and control groups (Figure 9a).

For IL-10 levels, all groups exhibited similar baseline levels (15–18 ng/L) at 0 dpi. By 7 dpi, IL-10 levels increased slightly across all groups, with no significant differences. At 28, 35, and 56 dpi, IL-10 levels were slightly elevated but showed minimalistic variation between groups (Figure 9b).

**Figure 10:**
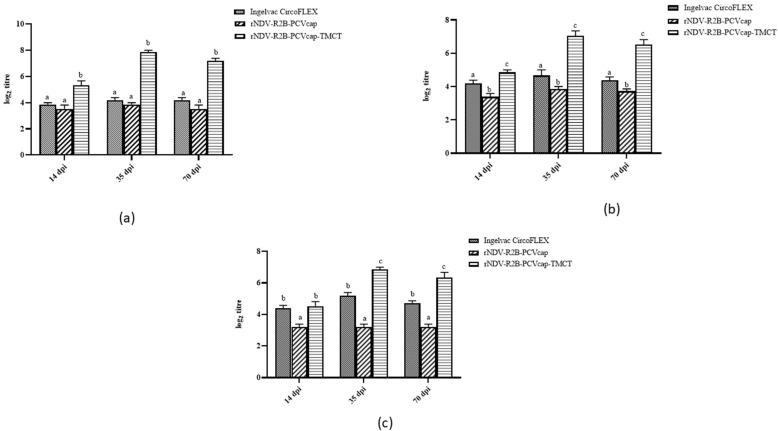
Neutralization antibody titres in the experimental pigs against PCV2 genotypes. Sera collected from vaccinated pigs at 14, 35 and 70 dpi were neutralized with (**a**) PCV2d genotype; (**b**) PCV2b genotype and (**c**) PCV2a genotype. Bars (Mean ± SE) indicate the representative data of a single experiment. Data with different small letters superscript indicate treatment effect (*p* < 0.05).

The virus neutralization and cross-neutralization assays revealed that the rNDV-R2B-PCVcap-TMCT group produced significantly higher neutralizing antibody titre (*p* < 0.05) against homologous and heterologous PCV2 genotypes PCV2d, PCV2b, and PCV2a, respectively, followed by Ingelvac CircoFLEX and rNDV-R2B-PCVcap groups on 14, 35, and 70 dpi (Figure 10). Virus-neutralizing antibody titre was the highest in the rNDV-R2B-PCVcap-TMCT group against PCV2d on day 35, which was significantly higher (*p* < 0.05) than groups rNDV-R2B-PCVcap and Ingelvac CircoFLEX, and it was significantly higher on 14 and 70 dpi as well. However, no significant difference (*p* > 0.05) was noticed in rNDV-R2B-PCVcap and Ingelvac CircoFLEX group against PCV2d on 14, 35, and 70 dpi. A similar trend was observed against PCV2b, with the highest titre showing in the rNDV-R2B-PCVcap-TMCT group, followed by Ingelvac CircoFLEX and rNDV-R2B-PCVcap. For the PCV2a genotype, there was a significant difference (*p* < 0.05) among the three groups on days 35 and 70, but the highest titre was observed in rNDV-R2B-PCVcap-TMCT, followed by Ingelvac CircoFLEX and rNDV-R2B-PCVcap groups. None to minimal neutralizing antibody titres were detected in control and rNDV-R2B groups on 14, 35, and 70 dpi.

## 4. Discussion

Following the global second genotypic shift of PCV2, there has been an increase in the virus’s virulence and a rise in vaccine failures, leading to greater economic losses in the swine industry. These losses are primarily due to reduced weight gain, reproductive failure, and immunosuppression [1]. A vector-based recombinant vaccine is a better approach to tackling ongoing vaccine failure and inducing long-lasting immune responses. The aim of this study was to generate two promising vaccine candidates using the mesogenic NDV R2B strain as a vector and expressing a modified synthetic cap gene of PCV2 [8,27]. Our research group has developed recombinant NDV-R2B and NDV-F strains as live viral vectors to deliver immunogenic genes of many important avian and non-avian viral diseases [19,28,29,30]. In one of the vaccine candidates, the transmembrane domain and cytoplasmic tail (TMCT) of the F gene of the NDV R2B strain were fused with the PCV2 cap gene. The recombinant vaccine candidates were stably expressed and maintained the integrity of the cap gene along with the TMCT domain in Vero cells and induced superior antibody responses in both mice and pigs.

The introduction of mutations (L59A, G191E, and K235Δ) into the PCV2 cap gene enhanced its stability, expression, and immunogenic properties, as was evident from a stronger immune response [31,32]. Further, the incorporation of transmembrane (TM) and cytoplasmic tail (CT) domains facilitated the efficient anchoring of the Cap protein on the cell surface and improved antigen presentation and uptake by antigen-presenting cells (APCs), ultimately leading to better T-cell activation and an enhanced immune response [33,34]. The TM domain ensures proper membrane localization and structural stability of therapeutic proteins, improving their accessibility to immune receptors and antibodies, which, in turn, enhances immune recognition [35]. Humoral immunity plays a pivotal role in the host defense against PCV2 infection, and this study demonstrated that both recombinant NDV-R2B-PCVcap-TMCT and NDV-R2B-PCVcap viruses elicited robust PCV2-specific and neutralizing antibody (NA) responses both in mice and pigs. Neutralizing antibodies are critical for preventing viral entry by binding to specific epitopes on the viral capsid, thereby blocking infection. High titers of these antibodies were strongly associated with reduced viral replication and less severe pathological lesions, highlighting their key role in controlling the virus [23,36]. Moreover, the humoral immune response elicited by PCV2 vaccine candidates is pivotal to clear PCV2 infection in pigs [25,37]. The vaccine candidate rNDV-R2B-PCVcap-TMCT elicited a strong humoral immune response in terms of IgG antibody specific to cap protein of PCV2 as well as neutralizing antibodies, which lasted throughout the experiment study period of 70 dpi in pigs. The observed decrease in PCV2 viral load and the corresponding histopathological improvements in vaccinated mice further underscore the protective efficacy of neutralizing antibodies [38,39,40]. The majority of available commercial PCV2 vaccines are based on the PCV2a genotype, but due to viral evolution, new genotypes have emerged [41], and the current dominating genotype is PCV2d. The currently available vaccines can reduce the infection caused by PCV2b and PCV2d genotypes besides PCV2a infection or viremia and transmission in experimental pigs [42,43], but as reported earlier, there may be chronic or subclinical PCV2 infection and the virus may not get completely cleared [25]. Hence, PCV2a-based commercial vaccines may provide incomplete cross-protection against the most prevailing genotype PCV2d [25,44]. The neutralizing and cross-neutralizing antibodies produced by the vaccine candidates were evaluated against common genotypes PCV2d, PCV2a, and PCV2b and it was observed that both rNDV-R2B-PCVcap-TMCT and rNDV-R2B-PCVcap groups showed promising neutralizing antibody titer against all the three genotypes, indicating their potential to protect the pigs against the commonly available genotypes.

The cellular immune response plays a pivotal role in the host’s defense against PCV2 infection, wherein both CD4+ and CD8+ T cells contribute to controlling viral replication and eliminating infected cells [45]. In the present study, the high antigen-specific T cell proliferation observed in the rNDV-R2B-PCVcap-TMCT group, followed by the rNDV-R2B-PCVcap group, suggests that these recombinant vaccines were effective in inducing a cellular immune response. The vaccines likely generated robust immunological memory, equipping the vaccinated animals with a rapid and potent response upon viral challenge [26,46]. Notably, the high CD4+ and CD8+ T cell populations and balanced CD4+/CD8+ T cell ratios in the rNDV-R2B-PCVcap-TMCT group in mice further indicated that this vaccine elicited the strongest cellular immune response. The elevated levels of these T cells, particularly post-challenge, suggest that a Th1-biased response is crucial for long-term protection [24]. In the case of PCV2 infection, neutralizing antibodies and cellular immune responses are essential immunological correlates of protection [47].

In response to PCV2 infection, immune cells produced the key cytokine IFN-γ where levels were inversely proportional to IL-10 levels. IFN-γ modulates the immune response and is critical for controlling PCV2 infection [48]. Specifically, IFN-γ production is essential for mounting a strong cell-mediated immune response to suppress viral replication, shedding, and PCV2-associated pathological lesions [40,43,49,50]. IFN-γ-secreting cells, which are pivotal for adaptive immunity against PCV2 [23,51], were significantly higher in the rNDV-R2B-PCVcap-TMCT and rNDV-R2B-PCVcap groups in both mice and pigs, indicating a strong Th1-type of immune response, which played a crucial role in viral clearance and long-term protection [52,53,54]. Induction of IFN-γ is an important immunological correlate of protection against PCV2 infection [47], which was also fulfilled by both rNDV-R2B-PCVcap-TMCT and rNDV-R2B-PCVcap vaccine candidates. The reduced IL-10 levels in the rNDV-R2B-PCVcap-TMCT and rNDV-R2B-PCVcap groups in both mice and pigs suggest a more balanced immune response, with reduced immunosuppression. Elevated IL-10 levels have been linked to immunosuppression in PCV2 infections [55,56], and limiting its production is beneficial for enhancing the antiviral immune response. Both the recombinant viruses rNDV-R2B-PCVcap-TMCT and rNDV-R2B-PCVcap showed neutralizing antibodies as well as cell-mediated immune responses against PCV2, indicating their protection potential in pigs.

Furthermore, the rNDV-R2B-PCVcap-TMCT vaccine demonstrated highest efficacy in reducing viral load in both the lung and inguinal lymph nodes of mice. This reduction was more pronounced in the rNDV-R2B-PCVcap group, followed by the Ingelvac CircoFLEX group, while the rNDV-R2B and control groups continued to exhibit significantly higher viral loads. This is particularly important as PCV2 targets lymphoid tissues, contributing to immunosuppression and respiratory disease. The decrease in viral load also coincided with the appearance of serum-neutralizing antibodies, underscoring the crucial role of humoral immunity in preventing viral entry and replication and the findings are consistent with previous reports [57,58].

PCV2 infection causes hallmark lesions such as lymphoid depletion and histiocytic replacement in lymphoid tissues, leading to immunosuppression [47]. The histopathological evaluation in mice revealed that the control and rNDV-R2B groups displayed significant histopathological lesions, such as lymphoid depletion, interstitial pneumonia, and hepatitis [59]. In contrast, the rNDV-R2B-PCVcap group, followed by the Ingelvac CircoFLEX group, exhibited varying degrees of protection against PCV2, with histopathological evidence showing mild to moderate tissue damage, respectively. Notably, the rNDV-R2B-PCVcap-TMCT group in mice displayed no signs of tissue damage, with the histological architecture of the lymphoid tissues remaining intact at both 7- and 14-dpc, indicating superior vaccine-induced protection against the PCV2-induced immunosuppression. The absence of PCV2 antigen in the inguinal lymph nodes of mice, as confirmed by immunohistochemistry, further supported the vaccine’s efficacy in preventing tissue damage and effectively controlling viral replication [54,60], highlighting the rNDV-R2B-PCVcap-TMCT vaccine’s robust capacity to mitigate the pathological effects of PCV2 infection.

In summary, the recombinant rNDV-R2B-PCVcap-TMCT virus with the modified cap gene and TMCT domain elicited the highest humoral and cellular immune responses against PCV2 infection in mice and pig models, demonstrated superior efficacy in controlling PCV2 replication, and prevented PCV2-induced pathology compared to the commercial vaccine. This comprehensive immune response, driven by both neutralizing antibodies against PCV2a, PCV2b, and PCV2d genotypes and Th1-type cellular immunity, underscores the potential of this recombinant virus in providing robust protection against PCV2 infection.

## Data Availability

All datasets generated for this study are included in the article.
